# Supplementary material for: Effects of Hot-Air Drying Temperatures on Quality and Volatile Flavor Components of Cooked Antarctic krill (Euphausia superba)
Source: Foods. 2025 Mar 31;14(7):1221. doi: 10.3390/foods14071221 (PMC11988459; doi:10.3390/foods14071221)
Supplement: Supplementary file 1 [file foods-14-01221-s001.zip › Table S1-sensory evaluation.pdf]

Table S1. Sensory evaluation criteria

Indexes	Criteria		
Appearance	Fragmentary with slight gray color	Slightly intact with shine color	Intact with fresh and bright red color
Smells	No seafood smell, serious peculiar smells	Slight seafood smell, slightly peculiar	Seafood smell, no peculiar smells
Texture	Loose	Slightly uniform/crisp	Tight and uniform
Grade	Bad (0-4)	General (5-7)	Excellent (8-10)
