# Supplementary material for: Effects of Hot-Air Drying Temperatures on Quality and Volatile Flavor Components of Cooked Antarctic krill (Euphausia superba)
Source: Foods. 2025 Mar 31;14(7):1221. doi: 10.3390/foods14071221 (PMC11988459; doi:10.3390/foods14071221)
Supplement: Supplementary file 1 [file foods-14-01221-s001.zip › Table S2.pdf]

Table S2. Stop time of different hot-air drying conditions for cooked Antarctic krill, and water content of dried Antarctic krill at the stop time.

	1	2	3	4
Stop time (s)	6083.00±3.00	3764.00±4.00	3720.00±8.00	2100.00±4.00
Water content (%)	26.45±0.03	26.15±0.02	24.20±0.01	22.71±0.01
