# Supplementary figures and images for: Effects of Hot-Air Drying Temperatures on Quality and Volatile Flavor Components of Cooked Antarctic krill (Euphausia superba)
Source: Foods. 2025 Mar 31;14(7):1221. doi: 10.3390/foods14071221 (PMC11988459; doi:10.3390/foods14071221)

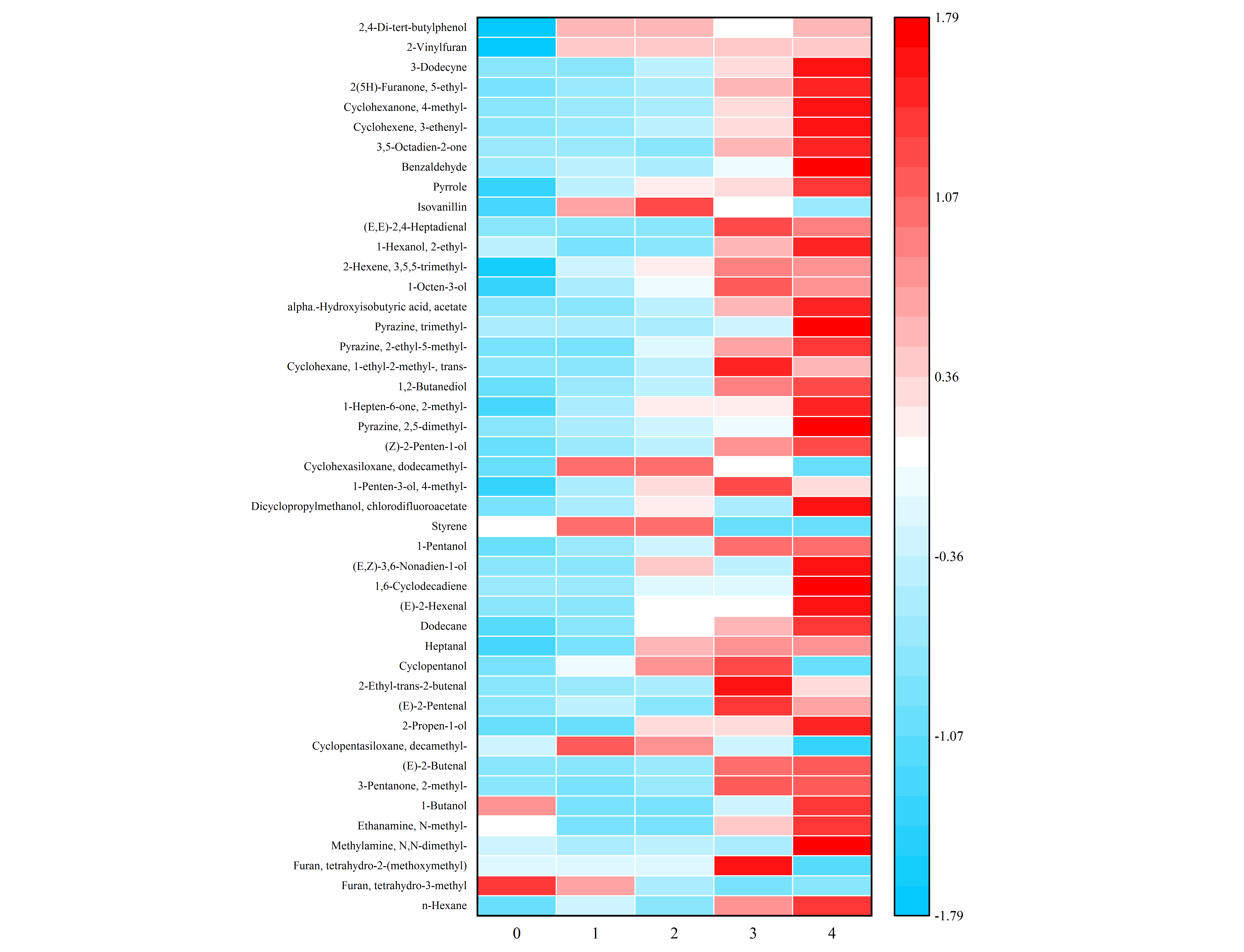

Supplement: Supplementary file 1 [file foods-14-01221-s001.zip › Figure S1 heatmap.jpg]
